# CPNE3 regulates the cell proliferation and apoptosis in human Glioblastoma via the activation of PI3K/AKT signaling pathway

**DOI:** 10.7150/jca.60049

**Published:** 2021-10-28

**Authors:** Dainan Zhang, Xiaoyin Wang, Xi Wang, Zemin Wang, Shunchang Ma, Chuanbao Zhang, Shaomin Li, Wang Jia

**Affiliations:** 1 Department of Neurosurgery, Beijing Tiantan Hospital, Capital Medical University, Beijing, China.; 2 Beijing Neurosurgical Institute, Capital Medical University, Beijing, China.; 3 Henan Key Laboratory of Neurorestoratology, The First Affiliated Hospital of Xinxiang Medical University, Weihui, Henan, China.; 4 Ann Romney Center for Neurologic Diseases, Department of Neurology, Brigham and Women's Hospital, and Harvard Medical School, Boston, MA 02115, USA.; 5 Chinese Glioma Genome Atlas Network (CGGA), Beijing, China.

**Keywords:** GBM, CPNE3, PI3K/AKT, cell proliferation, cell apoptosis

## Abstract

**Background:** Even with decades of intensive study, the signaling regulative network of the progression of Glioblastoma (GBM) remains unclear, a deeper understanding of the molecular crosstalk with pathways in GBM is needed to identify new potential targets for treatment. Copine-3 (CPNE3) was a member of a Ga2+ -dependent phospholipid-binding protein and was reported to play a role in multiple cancers.

**Methods:** To investigate the expression of CPNE3 in GBM, we applied bioinformatic analysis and clinical samples validation. Then the functional validation of carried out in commercially available glioma cell lines and nude mice model. Also, the GSEA analysis was used to identify the relevant pathways. The role of activated pathway was further validated by pharmacology method.

**Results:** We found that CPNE3 was significantly up-regulated in GBM when compared with adjacent normal tissues, and the overexpression of CPNE3 promoted cell proliferation and inhibiting cell apoptosis *in vitro* and *in vivo*. Also, the principal protein markers of PI3K/AKT pathway were found to be phosphorylated by CPNE3 over-expression, and pathway inhibitor, LY294002, alleviated the cell proliferation enhancement induced by CPNE3 over-expression.

**Conclusion:** Our results showed that the expression of CPNE3 promotes cell proliferation by inhibiting cell apoptosis via activating PI3K/AKT pathway. Thereby enhancing the progression of GBM, which suggest that CPNE3 may play as a tumorigenesis gene may become a promising potential therapeutic target for human GBMs.

## Introduction

GBM is the most common malignant primary tumor in the central nervous system 1. In the past decades, with the combination of standard treatment which includes surgical resection combined with chemotherapy and radiotherapy, the prognosis of GBM patients has importantly improved 2. However, for the sake of rapid tumor growth, the recurrent rate remains high and the outcomes of GBM patient is still poor. Therefore, the study underlies the mechanism of tumor proliferation and apoptosis as well as novel therapeutic strategies for GBM are needed 3, 4.

CPNE3 possess two C2Ds (C2D-A and C2D-B) and an A domain, C2Ds are responsible for Ca^2+^-dependent membrane-binding properties and A domain binds to proteins, it was reported to regulate molecular events at the interface of the cell membrane and cytoplasm 5. CPNE3 was also identified as a ligand of ErbB2 and plays a significant role in multiple carcinogenesis. For example, CPNE3 was reported to be a novel oncogene in non-small cell lung cancer (NSCLC), and promoted NSCLC metastasis via FAK signaling pathway. Also, the overexpression of CPNE3 in acute myeloid leukemia may play as an adverse prognostic biomarker. However, the role of CPNE3 in GBM is rarely reported.

In this study, we found that the expression level of CPNE3 was significantly higher in GBM tissues in TCGA dataset. To verify the potential function of CPNE3 in GBM pathology, we knocked down and overexpressed CPNE3 in GBM cell lines. Then the biological function and molecular mechanism of CPNE3 on proliferation and apoptosis were studied *in vitro* and vivo. What is more, we found the PI3K/AKT signaling pathway plays an important role in GBM, the relationship between CPNE3 and PI3K/AKT has also been explored. Our findings may shed a light on developing novel therapeutic strategies against GBM.

## Results

### The overexpression of CPNE3 in GBM

**Figure 1:**
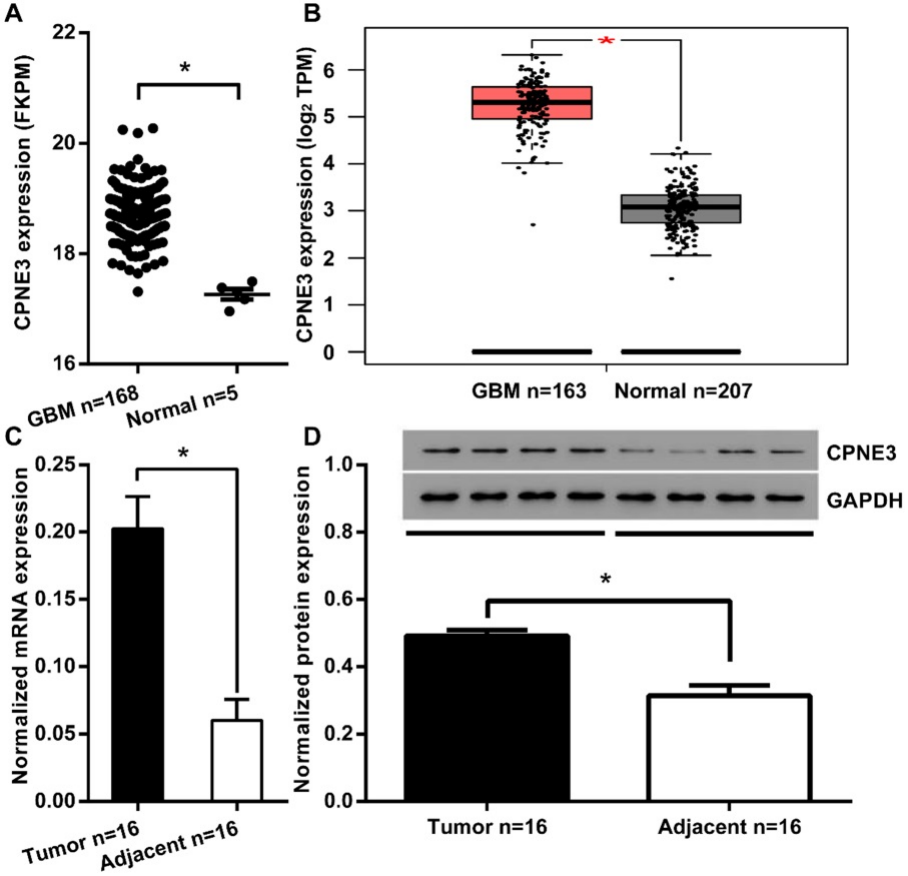
** CPNE3 is up-regulated in GBM tissues. (A)** The mRNA expression level of CPNE3 in GBM tissues (GBM n=168) is significantly higher when compared with adjacent normal tissues (Normal n=5) in the TCGA database. **(B)** The mRNA expression level of CPNE3 in GBM tissues (GBM n=163) is significantly higher when compared with adjacent normal tissues (Normal n=207) in GEPIA database. **(C)** Relative expression levels of CPNE3 mRNA in clinical GBM samples (Tumor n=16) and paired adjacent normal tissues (Adjacent n=16). **(D)** The representative 4 out of 16 blotting of 4 pairs clinical GBM samples (left) and adjacent normal tissues (right). Together with bar chart of normalized protein level of CPNE3 in the total 16 pairs of clinical samples. All data are expressed as mean ± SD. *p < 0.05 between indicated groups.

To investigate the function of CPNE3 in GBM, we first compared the expression of CPNE3 in GBM tumor tissues and adjacent normal tissues from TCGA/GEPIA dataset. We found that the mRNA expression of CPNE3 was significantly up-regulated in GBM tissues when compared to adjacent normal tissues (Fig. 1A, B). To further confirm this phenomenon, we collected 16 GBM samples with paired adjacent normal tissues in Tiantan hospital. Also, the mRNA and protein expression of CPNE3 were both found to be higher those in paired adjacent normal tissues (Fig. 1C, D). The bio-informative analysis together with our validation in clinical samples indicates that CPNE3 may play as tumorigenesis gene in GBM pathology.

### The expression and regulation of CPNE3 in GBM cell lines

**Figure 2:**
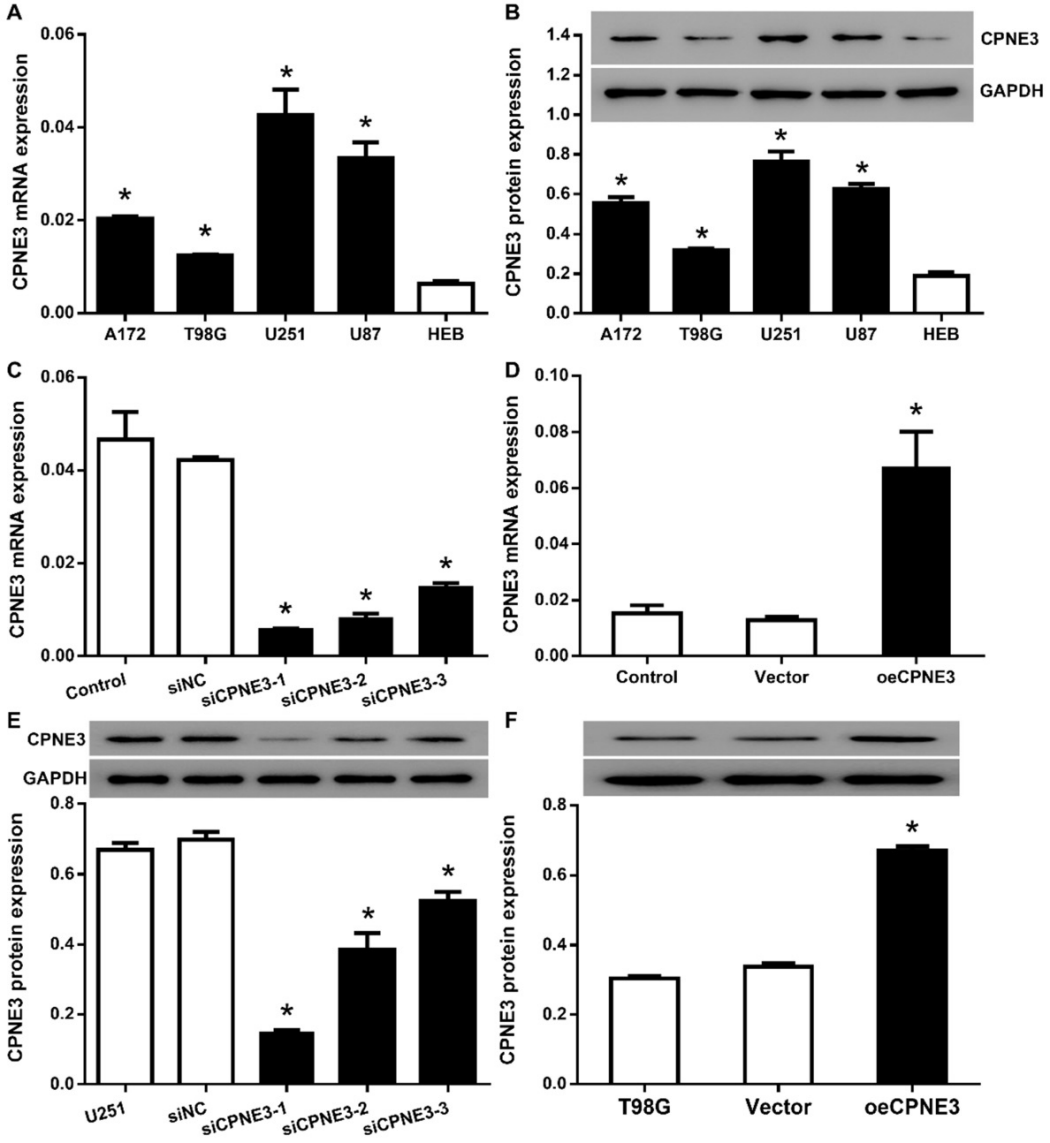
** The expression of CPNE3 was regulated in GBM cell lines. (A)** Relative expression levels of CPNE3 mRNA in GBM cell lines, including A172, T98G, U251, U87, compared with normal human glia cell line HEB. *p < 0.05 when compared to HEB group. **(B)** The representative blotting and bar chart of normalized protein level of CPNE3 in A172, T98G, U251, U87 and HEB cell lines. *p < 0.05 when compared to HEB group. **(C)** The mRNA expression of CPNE3 were downregulated in CPNE3 knockdown groups (siCPNE3-1, siCPNE3-2, siCPNE3-3) when compared to the blank control (U251) and negative control group (siNC). *p < 0.05 when compared to control groups. **(D)** The mRNA expression of CPNE3 were upregulated in CPNE3 overexpression group (oeCPNE3) when compared to the blank control (T98G) and negative control group (Vector), *p < 0.05 when compared to control groups. The representative blotting and the bar chart of normalized CPNE3 expression in CPNE3 knockdown U251 cells **(E)** and CPNE3 overexpressed T98G cells **(F)**. *p < 0.05 when compared to control groups.

In order to select proper cell model, we then analyzed the expression of CPNE3 in human glioma cell lines, A172, T98G, U251 and U87, as well as human glial cell line HEB. All the glioma cell line featured with a significantly higher CPNE3 mRNA (Fig. 2A) and protein (Fig. 2B) expression when compared with HEB cell line, and the glioma cell lines with the highest (U251) and the lowest (T98G) expression were selected for the CPNE3 knock-down and overexpression study.

Then, we attempted to transfer lentivirus particles packaged with 3 different siCPNE3 and all of those markedly reduced the mRNA and protein level of CPNE3 in U251 cells (Fig. 2C & E), siCPNE3-1 and siCPNE3-2 were selected for cell functional experiments due to a better knockdown efficiency. In contrast, lentivirus packaged with CPNE3 was transfected into T98G cells to induce CPNE3 overexpression (Fig. 2D & F).

### CPNE3 promotes GBM cells proliferation

**Figure 3:**
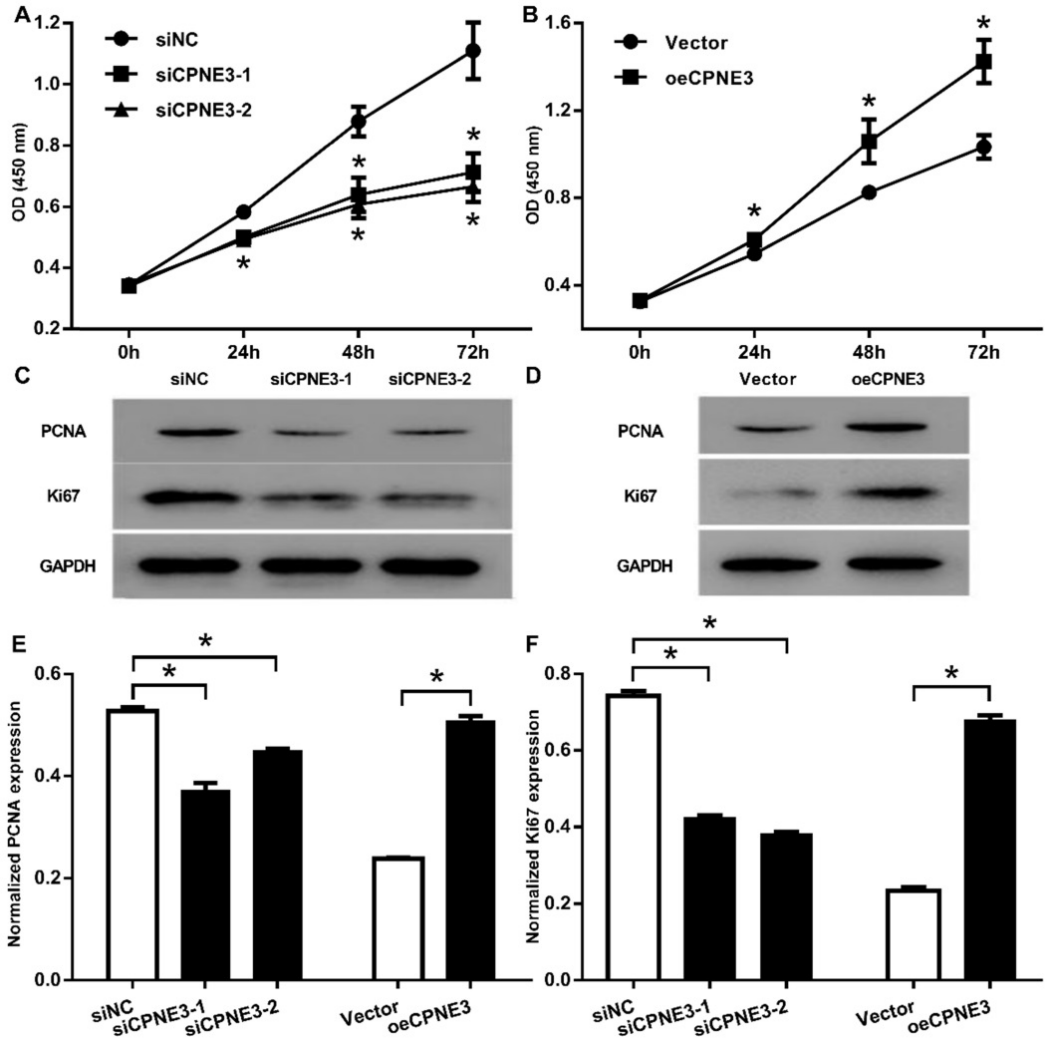
** CPNE3 promotes cell proliferation in GBM cell lines. (A)** CCK-8 assays indicated that U251 cells proliferation was suppressed after transfected with siRNA oligos targeting CPNE3, *p < 0.05 when compared to siNC group. **(B)** CCK-8 assays indicated that overexpression of CPNE3 promoted the cellular proliferation of T98G cells. The representative blotting expression of the PCNA and Ki67 in U251 cells of transfected with siCPNE3, *p < 0.05 when compared to Vector group. **(C)** and CPNE3 over-expressed T98G cells **(D)**. The bar chart showed the normalized expression of PCNA **(E)** and Ki67 **(F)** in CPNE3 suppressed U251 cells and CPNE3 overexpressed T98G cells. *p < 0.05 between indicated groups.

To investigate the role of CPNE3 on cell proliferation, we carried out CCK-8 assay and showed that, the down regulation of CPNE3 significantly inhibited the proliferation of U251 cells started from 24 h to 72 h (*p*<0.01, when compared with siNC treated cells, Fig. 3A), the overexpression of CPNE3 significantly promoted T98G cells proliferation 24h to 72 h (*p*<0.01, when compared with Vector treated cells, Fig. 3B). We also investigated the proliferation related protein level, no surprise, the Proliferating Cell Nuclear Antigen (PCNA) and Ki67 was significantly down-regulated in CPNE3 knock-down group (Fig. 3C), and significantly enhanced in CPNE3 over-expressed group (Fig. 3D). These results suggested that the CPNE3 plays a significant role in proliferation procedure of GBM cells.

### CPNE3 suppresses GBM cells apoptosis

**Figure 4:**
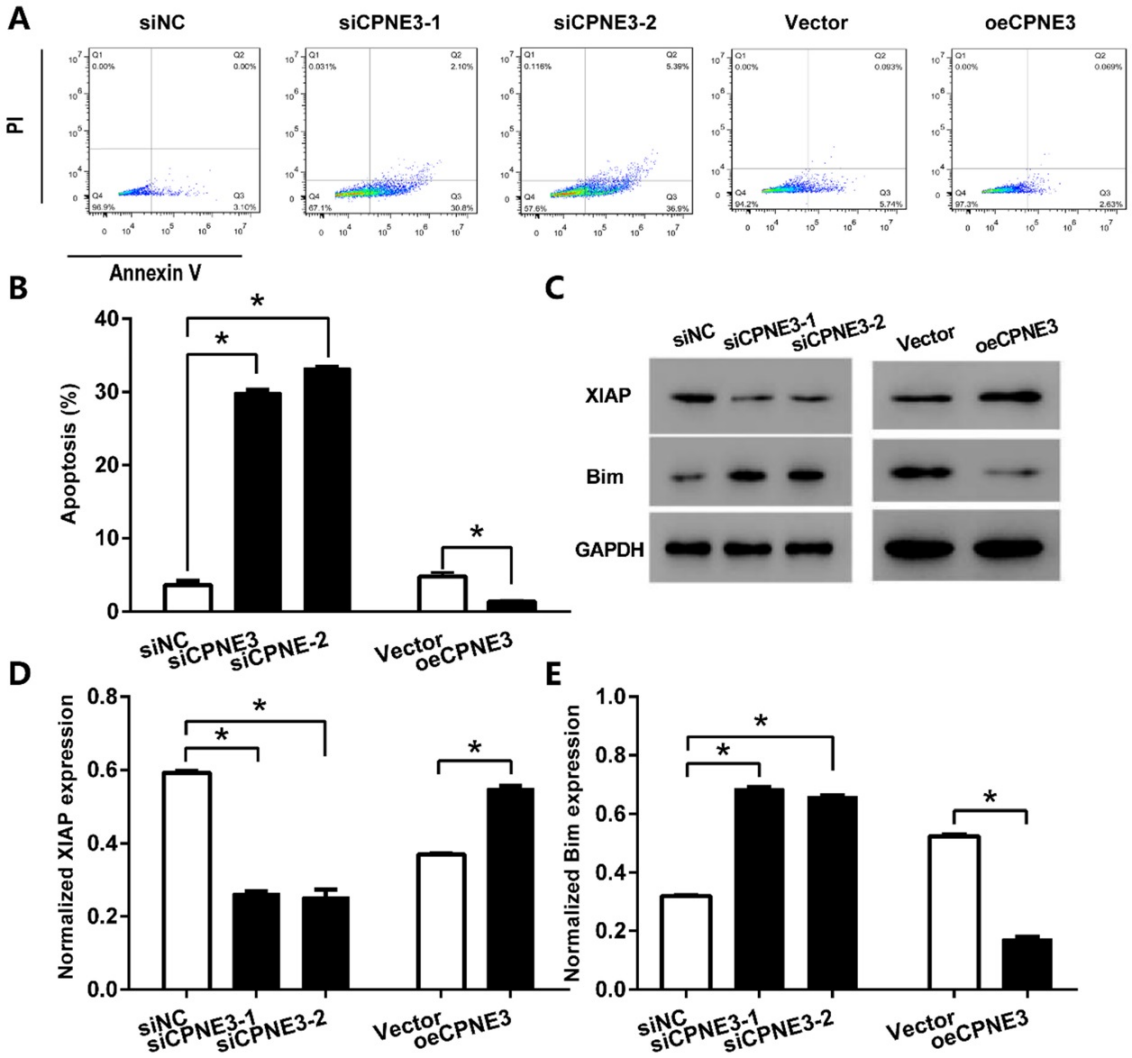
** CPNE3 suppresses GBM cells apoptosis. (A)** The U251 cells transfected with siCPNE3 (siCPNE3-1 and siCPNE3-2) and the T98G cells transfected with overexpression of CPNE3 (oeCPNE3) were stained and analyzed by flow cytometry. **(B)** The percentage of apoptosis cells were presented as mean ± SD. **(C)** The representative blotting of apoptosis related proteins XIAP and Bim, while the expression of CPNE3 were knockdown or overexpressed. **(D)** The bar chart showed the normalized expression of XIAP was inhibited by siCPNE3 and enhanced by CPNE3 over-expression. **(E)** The bar chart showed the normalized expression of Bim was enhanced by siCPNE3 and inhibited by CPNE3 over-expression. *p < 0.05 between indicated groups.

We further detected the effect of CPNE3 on apoptosis in GBM cell lines by using flow cytometric. With staining of Annexin V-FITC, our data showed that the percentage of apoptotic cells in U251 cells transfected with siCPNE3 was significantly enhanced (29.87 ± 0.72% for siCPNE3-1 and 33.22 ± 0.89% for siCPNE3-2, *p*<0.05 when compared with 3.58 ± 0.98% in negative control group). On the other hand, the percentage of apoptotic cells in T98G cells was significantly decreased in CPNE3 up-regulated group (1.40 ± 1.08%, *p*<0.05 when compared with 4.74 ± 0.94% in Vector treated group,) (Fig. 4A & B). We also analyzed the expression of apoptosis inhibitor protein X-linked inhibitor of apoptosis protein (XIAP) and apoptosis regulator protein Bim by western blotting technique (Fig. 4C), the results showed that siCPNE3 treatment in U251 cells inhibited the expression of XIAP, and promoted the expression of Bim. While the overexpression of CPNE3 in T98G cells showed an opposite result. ALL the above results suggest that CPNE3 affects the viability and proliferation of GBM cells at least in part by regulating cell apoptosis.

### Analysis of CPNE3-associated pathways

**Figure 5:**
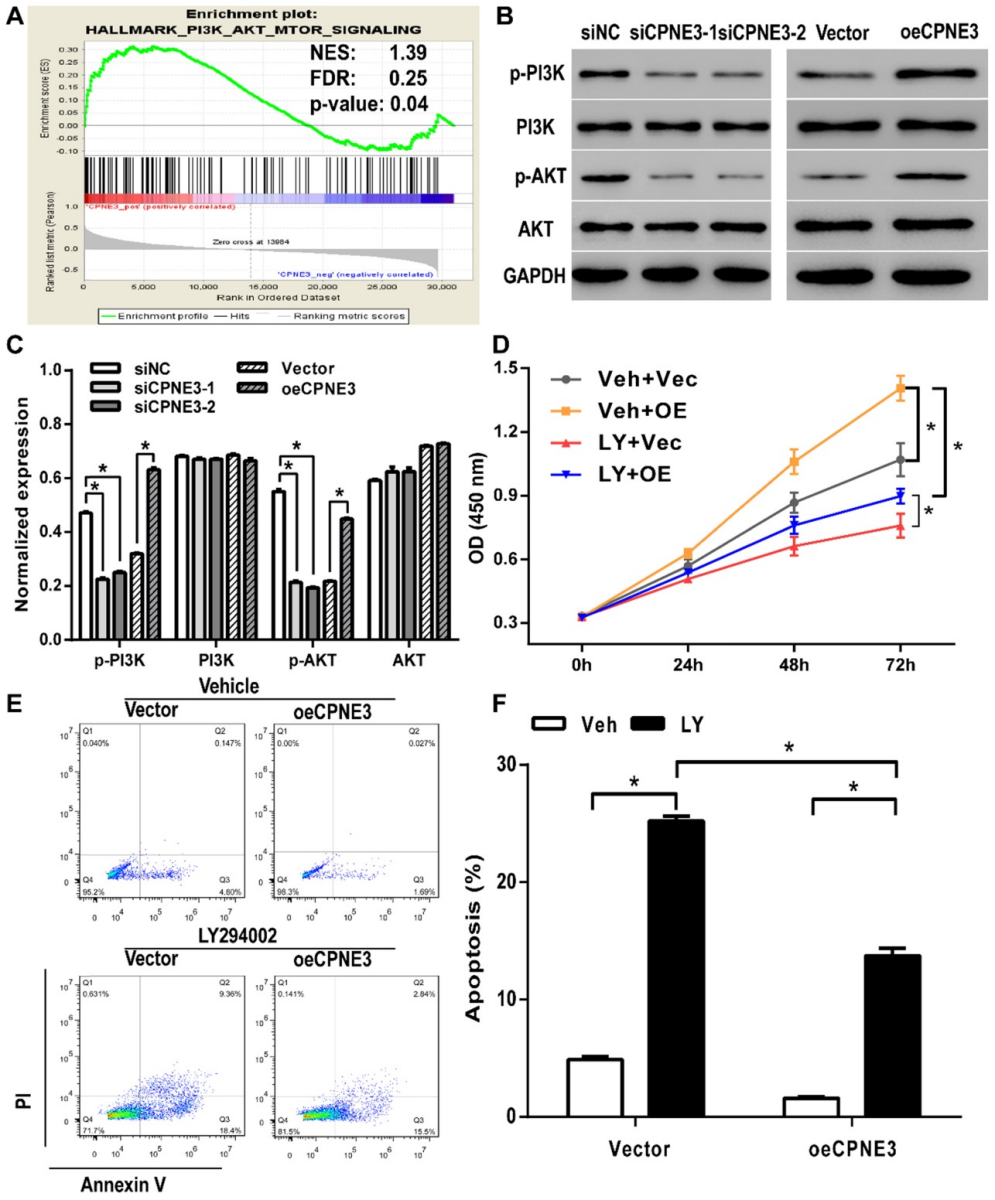
** PI3K/AKT signaling pathway was involved in CPNE3 induced GBM cell proliferation and apoptosis modulation. (A)** GSEA suggested that CPNE3 achieved the modulatory effect via the PI3K/AKT signaling pathway activation. **(B)** The representative blots of the key proteins of PI3K/AKT pathway in CPNE3 down-regulated U251 cells (siCPNE3-1 and siCPNE3-2) and CPNE3 overexpressed T98G cells (oeCPNE3). **(C)** The bar chart showed the expression of CPNE3 positively correlated with PI3K/AKT phosphorylation (p-PI3K and p-AKT), while the total expression of PI3K/AKT have no significant change (PI3K and AKT). **(D)** The over-expression of CPNE3 promotes GBM cell proliferation (Veh + OE, yellow squares with line) in T98G cells when compared with negative control group (Veh + Vec, grey circles with line); this enhancement effect that induced by CPNE3-overexpression was inhibited by PI3K inhibitor, LY294002 (LY + OE, blue downward circles with line). **(E & F)** The CPNE3-overexpression (oeCPNE3) induced a significant suppression of cell apoptosis in T98G cells, while PI3K inhibitor LY294002 (LY294002, black bars) reversed the apoptosis inhibition effect in both CPNE3-overexpression (oeCPNE3) and CPNE3-wildtype (Vector) cells. The cell apoptosis was analyzed by flow cytometry. The percentage of apoptosis cells are presented as mean ± SD. *p < 0.05 between indicated groups.

Gene set enrichment analysis (GSEA) was performed to evaluate pathways that were associated with CPNE3 expression in the TCGA GBM samples. The results revealed that CPNE3 expression was positively correlated with PI3K-AKT-mTOR signaling pathway (Fig. 5A). The PI3K/AKT pathway plays an essential role in cell proliferation in various types of cancer, which implied that CPNE3 may modulate the proliferation and apoptosis of GBM by affecting PI3K/AKT pathway. Hence, we performed western blotting to detect whether the PI3K/AKT pathway was involved in the adjustment of proliferation under the influence of CPNE3. As shown in Fig. 5B&C, the expression of phosphorylated PI3K and phosphorylated AKT in U251 cells that transfected with siRNA-CPNE3 was decreased, an opposite result was obtained in CPNE3 up-regulated T98G cells, while no change was observed in the expression of total PI3K and total AKT in both treatments.

### CPNE3 regulates the cell proliferation and apoptosis via the PI3K/AKT pathway

To further explore the mechanism of CPNE3 involved regulatory effect, we applied PI3K inhibitor LY294002 to suppress the PI3K/AKT pathway activation. As shown in Fig. 5D, CCK8 assays revealed that LY294002 treatment inhibited CPNE3 over-expression mediated cell proliferation enhancement in T98G cells. Also, the flow-cytometric analysis with Annexin V-FITC staining showed that the percentage of apoptotic cells in T98G cells was significantly increased by LY294002 treatment (18.77 ± 0.57%, *p*<0.05 when compared with 4.90 ± 0.38% in vehicle treated group), and the apoptosis inhibition effect of CPNE3 overexpression was also alleviated by LY294002 application (12.05 ± 1.18% in LY294002 treated CPNE3 overexpressed cells, *p*<0.05 when compared with 1.59 ± 0.20% in vehicle treated CPNE3 overexpressed cells) (Fig. 5E & F). These results suggest that CPNE3 enhances the proliferation and depresses the apoptosis of GBM cells by activating the PI3K/AKT pathway.

### Down-regulation of CPNE3 suppresses tumorigenicity of U251 cells *in vivo*

**Figure 6:**
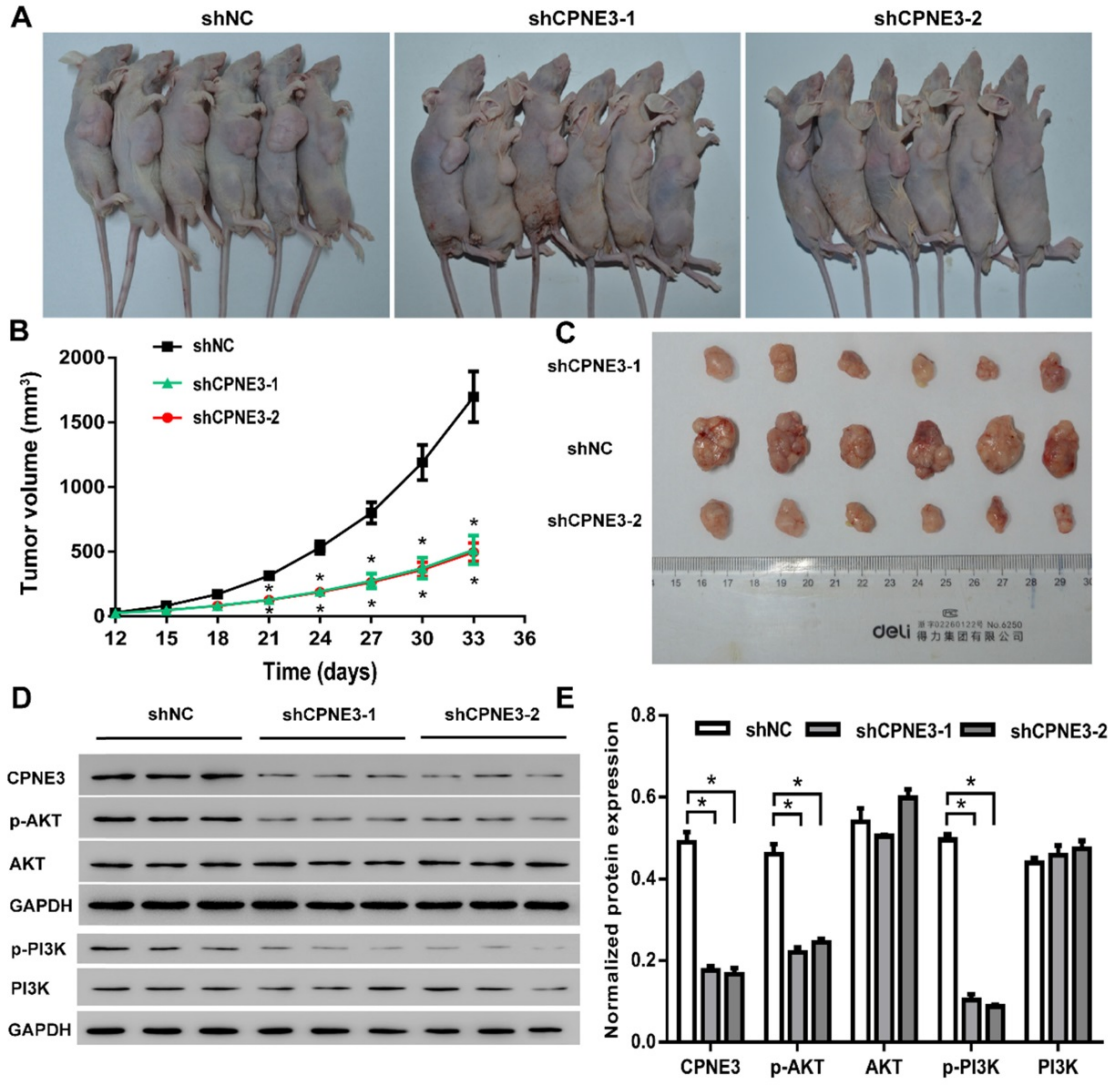
** CPNE3 promotes GBM tumor growth in nude rat xenograft tumor model. (A)** Images of shCPNE3 and shNC treated xenograft tumors in nude rats. **(B)** Tumor size was measured from day 12 to day 33 post implantation at an interval of 3 days; and the tumor volume was calculated. *p < 0.05 when compared to shNC group. **(C)** The rats were euthanized 33 days post implantation, and the xenograft tumors were collected and weighted in displayed three groups. **(D and E)** Western blot assays showed that the knock-down of CPNE3 correlated with a depressed PI3K/AKT activation, without a significant change of total PI3K/AKT expression. *p < 0.05 between indicated groups.

In order to investigate the effects of CPNE3 on glioma growth *in vivo*, a xenograft nude mouse model was established. A stable CPNE3 knockdown U251 cell line was implanted in nude mice and showed significantly slower growth rate than those in control group (Fig. 6A & B). Additionally, the tumors collected from mice injected with CPNE3 down-regulated U251 cells were significantly smaller than those in control group (Fig. 6C). Immunofluorescence staining was performed to detect the expression of PCNA, Ki67 in the xenograft tumor tissues. As shown in Supplementary Fig. S1, the fluorescence signal of PCNA and Ki67 were significantly decreased in tumor tissues from CPNE3 knock-down group. In HE staining study, the cell density was found to be lower, as well as the nuclei were shrinked in CPNE3 knock-down group (Supplementary Fig. S2). In line with our previous result, the expression of p-PI3K and p-AKT was significantly suppressed in CPNE3 knockdown animal while total PI3K and AKT expression had no significant change (Fig. 6D & E).

## Discussion

GBM is the most common malignant brain tumor, and is among the most lethal of all cancers 6, 7. The current standard treatment for GBM, including surgical operation, radiological and chemical therapy, were able to eliminate the majority of tumor cells, however recurrence were still inevitable. The features of rapid tumorigenesis may involve many signaling pathway, for example, previous study showed EGFR/erbB2 oligomerization affects the PI3K/AKT pathway to inhibit GBM cell proliferation 8. The complex regulatory network indicates comprehensive study of GBM cell proliferation mechanism remains to be elucidated.

CPNE3 is a functional protein in many diseases, especially in cancers, such as non-small cell lung cancer, breast cancer, prostate cancer, colorectal cancer and acute myocardial infarction 9-13. However, the role of CPNE3 in GBM remains poorly understood. Our results showed that CPNE3 was significantly up-regulated in human GBM tissues and promoted the cell proliferation by depressing the cell apoptosis *in vitro* and *in vivo*, we also found this regulatory effect was correlated to PI3K/AKT signaling pathway activation. Our data indicates that CPNE3, as well as the PI3K/AKT pathway has the potential to be a novel and valuable therapeutic target for human GBM. What is more, overexpressed CPNE3 decreased the cells apoptosis and inhibited intrinsic apoptosis pathway, as declined expression of Bim and elevated expression of XIAP were observed.

Previous studies have demonstrated that the PI3K/AKT pathway played a key role in modulating cell proliferation and apoptosis 14, 15. In glioma studies, multiple evidences revealed that the activation of PI3K/AKT pathway could enhance cell proliferation in a mTOR dependent or independent way 16-18. In primary breast tumors, high CPNE3 RNA levers significantly correlate with ERBB2 amplification 10. Other studies suggest that binding of CPNE3 to ERBB2 is increased when Jab1 is overexpression in SKBR3 breast cancer cells. Furthermore, two ERBB2 downstream signaling proteins p-PI3K and p-AKT were also activated by Jab1 overexpression in these cells 19. However, as far as we could reach, no convincing evidence showed the regulatory effect of CPNE3 on PI3K/AKT pathway in GBM. In our study, we blocked the PI3K/AKT pathway by using LY294002 in T98G cells and found that the cell proliferation promotion and cell apoptosis inhibition mediated by CPNE3-overexpressing was reversed. Also, in the nude mice xgenograft model, the knockdown of CPNE3 expression had a strong correlation with depressed expression of phosphorylated AKT, while total expression of AKT had no significant change. Our data provided evidence suggesting that CPNE3 may affect glioma cell proliferation and apoptosis by activating the PI3K/AKT signaling pathway.

In summary, our study showed that CPNE3 was up-regulated in GBM, and aberrant expression of CPNE3 can influence cell proliferation and apoptosis of GBM through PI3K/AKT pathway activation. Therefore, CPNE3 may play a pivotal role in GBM progression and could be studied as a novel potential therapeutic target for GBM treatment. However, we have no in-depth study was conducted on the mechanism, which will be discussed in the following studies.

## Materials and Methods

### Cell lines and cell culture

Human GBM cell lines (A172, T98G, U251 and U87), human astrocytes cell line HEB and human HEK293T cell line were obtained from American Type Culture Collection (ATCC, USA). A172, T98G, U251, U87, HEB, HEK293T cells were cultured in DMEM (Hyclone, USA) supplemented with 10% heat-inactive FBS, 100 U/mL penicillin and 100 μg/mL streptomycin. Cells were incubated in a 5% CO2 humidified incubator at 37 °C. According to cell demands, the medium was refreshed in regular intervals.

### GBM sample collection

The GBM samples that used for expression level assay were collected from patients who underwent surgery at Tiantan Hospital, which were immediately frozen in liquid nitrogen after tumor resection. Informed consent of patients was obtained as approved by Ethics Committee of Beijing Tiantan Hospital, Capital Medical University (KY 2018-052-01).

### Lentiviral infection

Three siRNA (siRNA-1 (788-806): GGTGGAGTGTTATGATTAT; siRNA-2 (1397-1415): GCAGACAGCTTCTCAATAT; siRNA-3 (1465-1483): CCAGACAAGCTATAGTTAA) were synthesized and cloned in pLVsiRNA. Then, to construct the overexpression plasmids, the coding sequence of human gene were synthesized and subcloned into the pcDNA3.1 lentivirus vector. Together with package plasmids, the siRNA-expressing and overexpressing constructed plasmids were transfected into 293T cells. The cells (with 80% of confluence) were infected with lentivirus supernatants, after 72h infection, the expression was determined using q-PCR and Western Blot.

### CPNE3 knockdown in xenograft mice model

CPNE3 knockdown was generated in U251 cells with pLKO.1 -puro lentiviral vectors expressing shRNA against CPNE3 gene products.

- shCPNE3-1 (788-806): GGTGGAGTGTTATGATTAT;
- shCPNE3-2 (1397-1415): GCAGACAGCTTCTCAATAT;
- shCPNE3-2 (1465-1483): CCAGACAAGCTATAGTTAA.

Oligonucleotides were amplified by PCR using the T4 DNA Polymerase (Thermo), PCR products containing shCPNE3-1, shCPNE3-2, shCPNE-3 and non-silencing shNC (controls) were subcloned into the pLKO.1-puro vectors via EcoR I and AgelI restriction sites.

### Quantitative Real-time PCR (qPCR)

Total RNA of suspension cultured all cells were isolated by using the RNAiso Plus kit (invitrogen). mRNA was transcribed into cDNA with OneScript Plus Reverse Transcriptase kit (Fermentas). q-PCR was performed using SYBR Green PCR MasterMix kit (Thermo) on Real-time System (ABI). Primer sequences are as follows:

- CPNE3: F 5'-CATTGTAGAGGCGTATCG-3', R 5'-CCATCACCATCCAGAAAC-3';
- GAPDH: F 5'-AATCCCATCACCATCTTC-3', R 5'-AGGCTGTTGTCATACTTC-3'.

The amount of PCR product for CPNE3 was normalized with the amount of GAPDH product to estimate the amount of variation between samples.

### Western Blot

Cells were lysed in RIPA lysis buffer containing 1 mM phenylmethanesulfonyl fluoride (PMSF). Total protein lysates (25 μg) were fractionated by 10% sodium dodecyl sulfate-polyacrylamide gel electrophoresis, transferred to PVDF membranes, and blocked at room temperature for 1 h with 5% nonfat dry milk and followed by overnight incubation with the anti-CPNE3 antibody (1:500, Abcam, Ab236606), anti-PCNA antibody (1:1000; Cell Signaling Technology, #13110), anti-ki67 antibody (1:500, Abcam, Ab16667), anti-XIAP antibody (1:1000, Abcam, Ab28151), anti-Bim antibody (1:1000, Abcam, Ab7888), anti-p-P13K antibody (1:1000, Abcam, Ab182651), anti-P13K antibody (1:1000, Abcam, Ab133595), anti-AKT antibody (1:1000, Cell Signaling Technology, #9272), anti-p-AKT antibody (1:2000, Cell Signaling Technology, #4060), anti-GAPDH antibody (1:2000, Cell Signaling Technology, #5174). An HRP-conjugated secondary antibody (1:1000, Beyotime) was applied for 1h at room temperature. The intensity for each protein band was corrected by the intensity of the GAPDH band and was normalized to facilitate comparisons.

### Cell proliferation assay

The cells viability was assessed by Cell Counting Kit-8 (CCK8) (SAB) according to the manufacturer's protocol. In brief, for CCK8 assay, cells were seeded in 96- well plates (3×10^3^ cells/well) and then cultured at 37 ℃ in a humidified atmosphere with 5% CO_2_. After 12h, 24h, 48h and 72h incubation, respectively, 10 μl CCK8 solution was added into each well. The plates were incubated for an additional 1h before detecting the absorbance value at 450 nm wavelength with a microplate reader (Perlong, Beijing).

### Cell apoptosis assay

For cell apoptosis assay, cells were seeded in 6-well plates and transfected, then the dual-staining of FITC-conjugated annexin V and propidium iodide (PI) was performed as follow. At 48h post-transfection, the cells, including floating cells, were harvested and washed twice with 4 °C PBS, then resuspended in binding buffer (10 mM HEPES/NaOH, 140 mM NaCl, 2 mM KCl). Annexin V was cultured for 15 mins in the dark. Cells were then washed again, centrif uged and resuspended in binding buffer. Before flow cytometric analysis, PI was added to each sample. The apoptotic cells were quantified by cytoFLEX system, cells labeled Annexin V +/PI- were early apoptotic cells.

### Nude mice xenograft study

The U251 cells were resuspended in serum-free DMEM medium at a concentration of 5 × 10^7^ cells/mL. Eighteen male nude mice were randomly assigned to three groups, and each mouse was inoculated with 0.1 mL of cell suspension in the right axillary sub cutis. The length and width of the tumor was measured weekly using a vernier caliper, and the tumor size was calculated as volume (mm^ 3^) = 0.5 × length (mm) × width^2^ (mm^2^). The mice were euthanized 5 weeks later, the tumors were collected and weighed, and the growth curve was calculated.

### Immunofluorescence

The xenograft tumor tissues were incised into small pieces, normally with a size of 1.5 cm×1.5 cm x 0.3 cm, and prepared by paraffin embedding. Generally, 5 µm sections were air-dried for 2h at room temperature. Sections were washed three times in PBS and blocked in 2% BSA, 0.1% Tween20 in PBS at RT. Then, sections were washed three times in PBS and incubated with primary antibodies, Ki-67 (Abcam, Ab15580) and PCNA (Abcam, Ab18197), at 4 °C overnight. The following day, sections were washed three times in PBST (0.05% Tween20 in PBS) and the Alexa Fluor 488 secondary antibodies (1:500, Byotime, A0423) were used. DAPI (1:500, Byotime, C1002) were added per section and incubated for 1h in the dark. All images were taken using a Fluorescence microscope (NIKON, ECLIPSE Ni).

### Histology

The xenograft tumor tissues were fixed and followed by paraffin embedding. Overall, 5 µm sections were air-dried for 2h at room temperature. Tissue sections were deparaffinized, rehydrated, and histologically stained with hematoxylin and eosin (H&E), All images were taken using a microscope (NIKON, ECLIPSE Ni).

### Gene set enrichment analysis

We used Gene Set Enrichment Analysis (GSEA v2.0, available online: http://www.broad.mit.edu/gsea/) to analyze the association between expression of CPNE3 and biological processes/pathway, phenotypes. Pre-defined gene set was obtained from the Molecular Signatures Database, MSigDB (http://software.broadinstitute.org/gsea/msigdb). Samples from the TCGA datasets were divided into high- or low- CPNE3 expression groups using the median as the cutoff. Default settings were used and thresholds for significance were determined by permutation analysis (1000 permutations). False Discovery Rate (FDR) was calculated. A gene set is considered significantly enriched when the FDR score is <0.25.

### Statistical analysis

Data was presented as mean of triplicates ± standard deviation (SD). Differences between groups were established by repeated-measures ANOVA followed by Bonferroni test and the Student's *t*-test. *p* values < 0.05 were considered significant.

## Supplementary Material

Supplementary figures.
