# Supplementary material for: CPNE3 regulates the cell proliferation and apoptosis in human Glioblastoma via the activation of PI3K/AKT signaling pathway
Source: J Cancer. 2021 Oct 28;12(24):7277–86. doi: 10.7150/jca.60049 (PMC8734413; doi:10.7150/jca.60049)

Figure S1. Representative images of PCNA and Ki67 staining of xenograft tumors tissues (400X).

PCNA SiNC

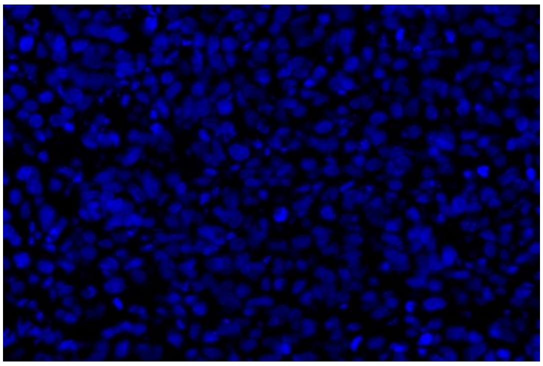

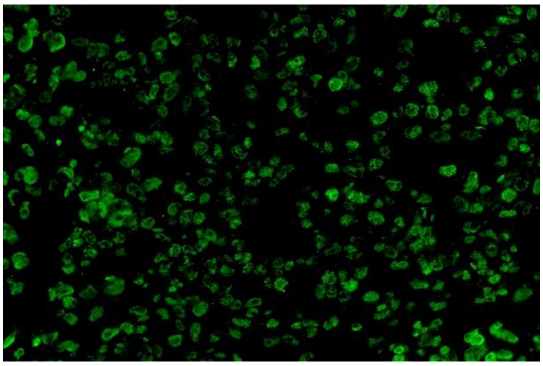

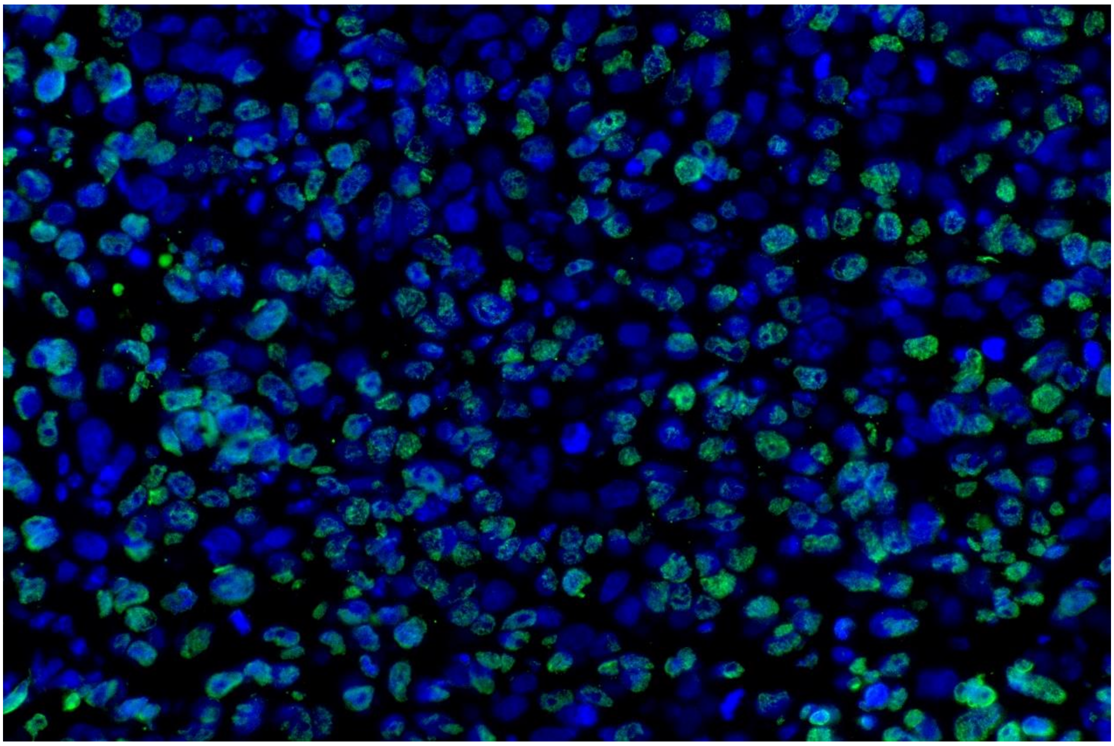

PCNA siCPNE3-1

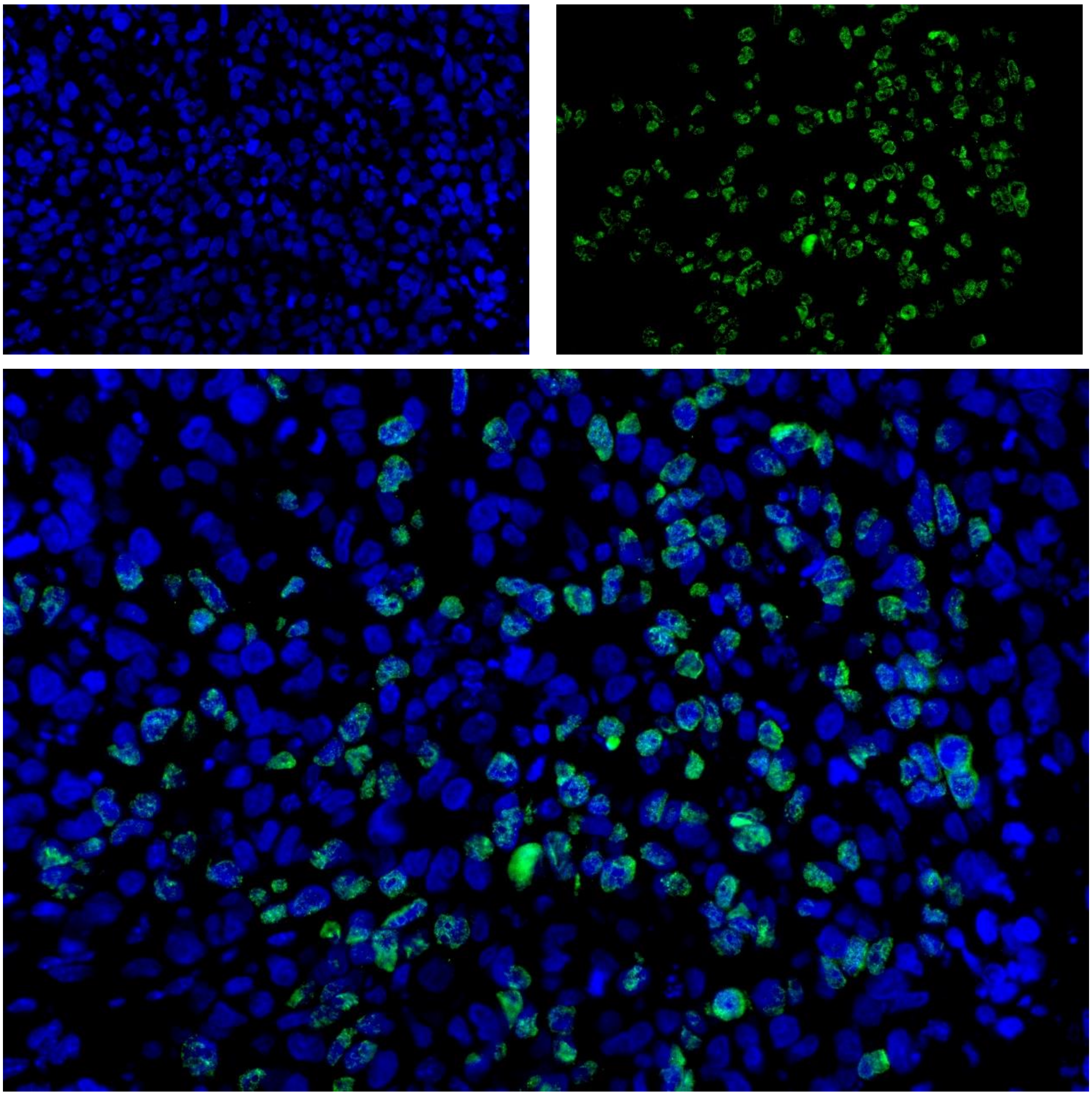

PCNA siCPNE3-2

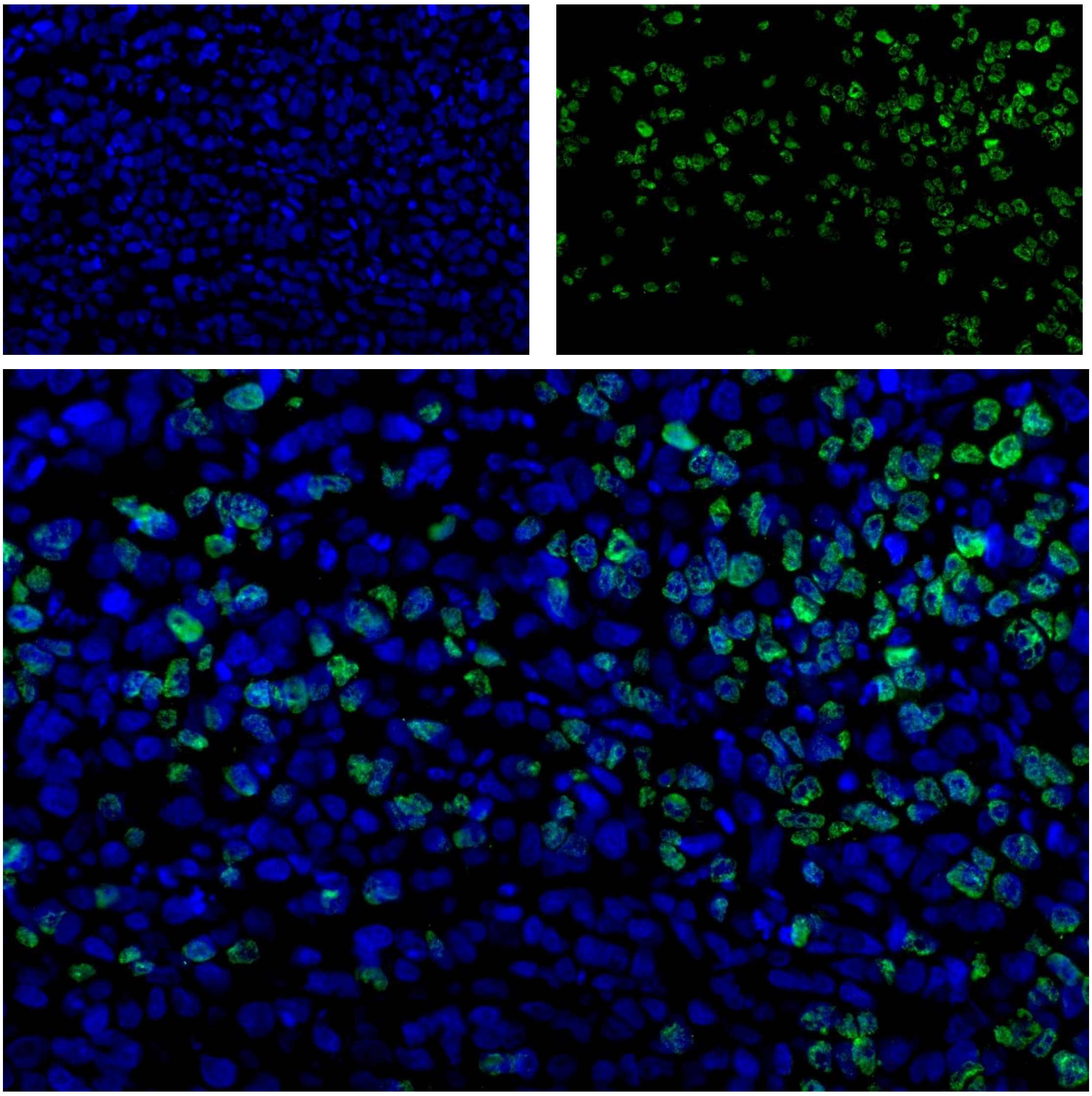

Ki67 siNC

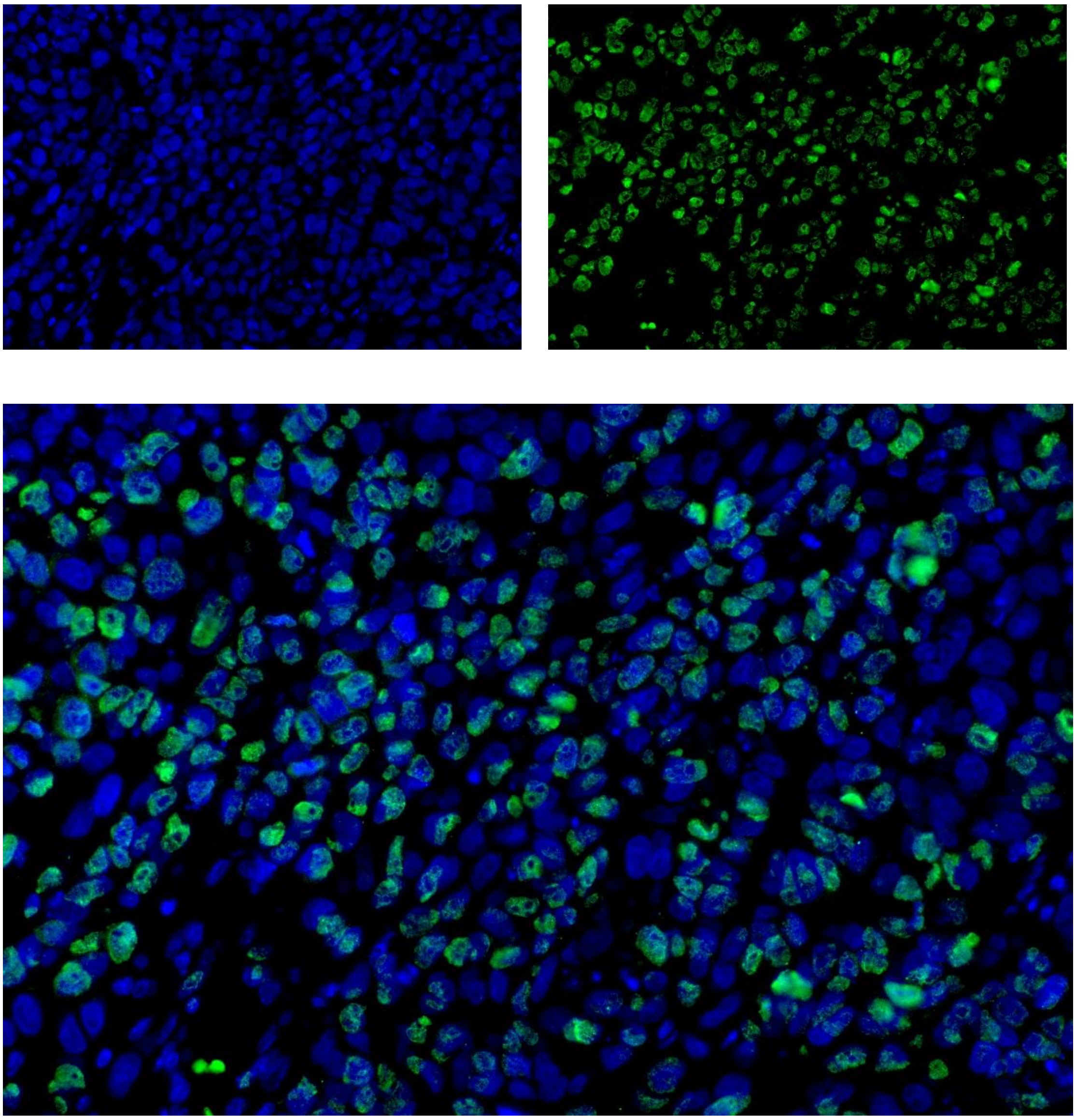

Ki67 siCPNE3-1

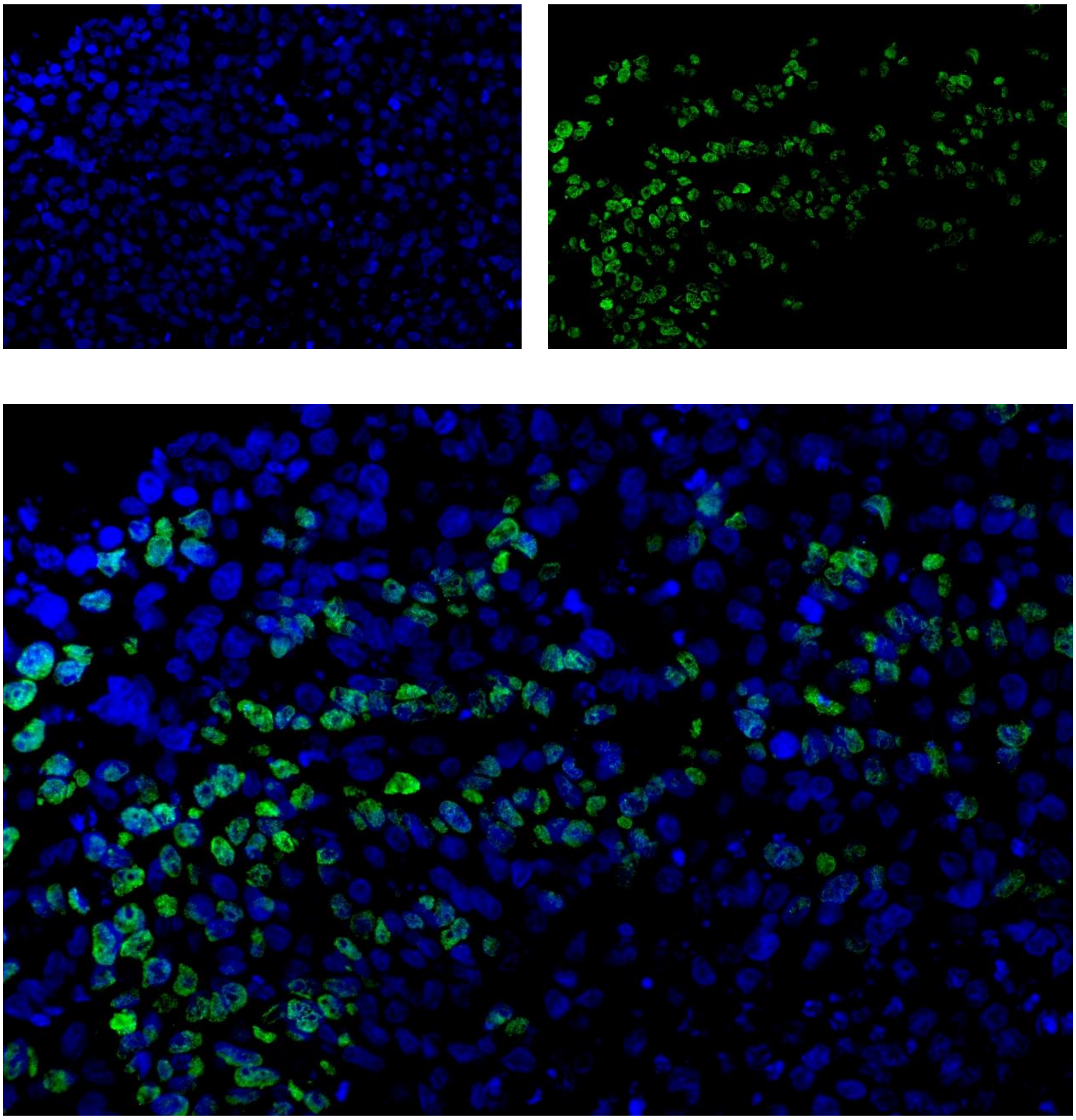

Ki67 siCPNE3-2

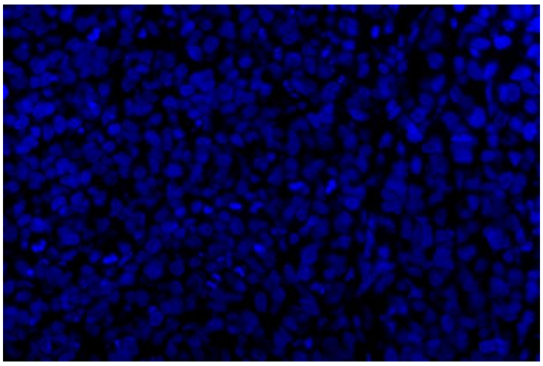

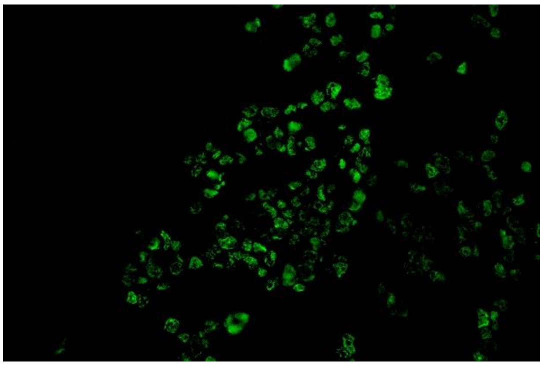

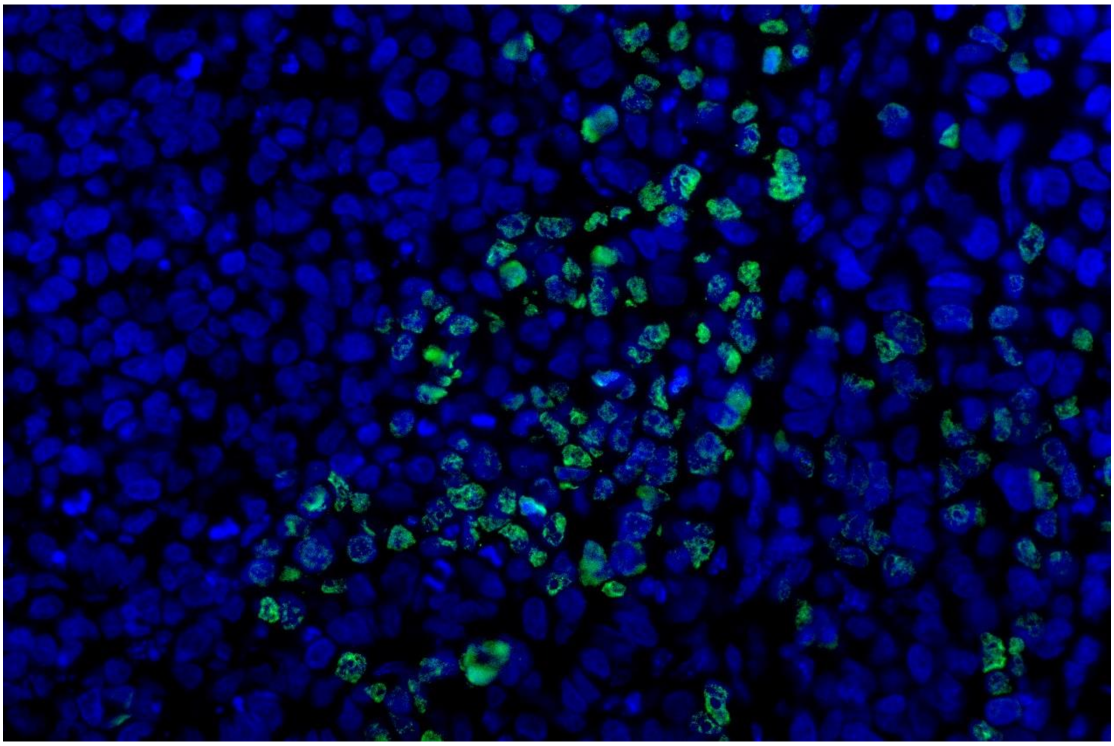

Figure S2. Representative images of HE staining of xenograft tumors tissues.

siNC

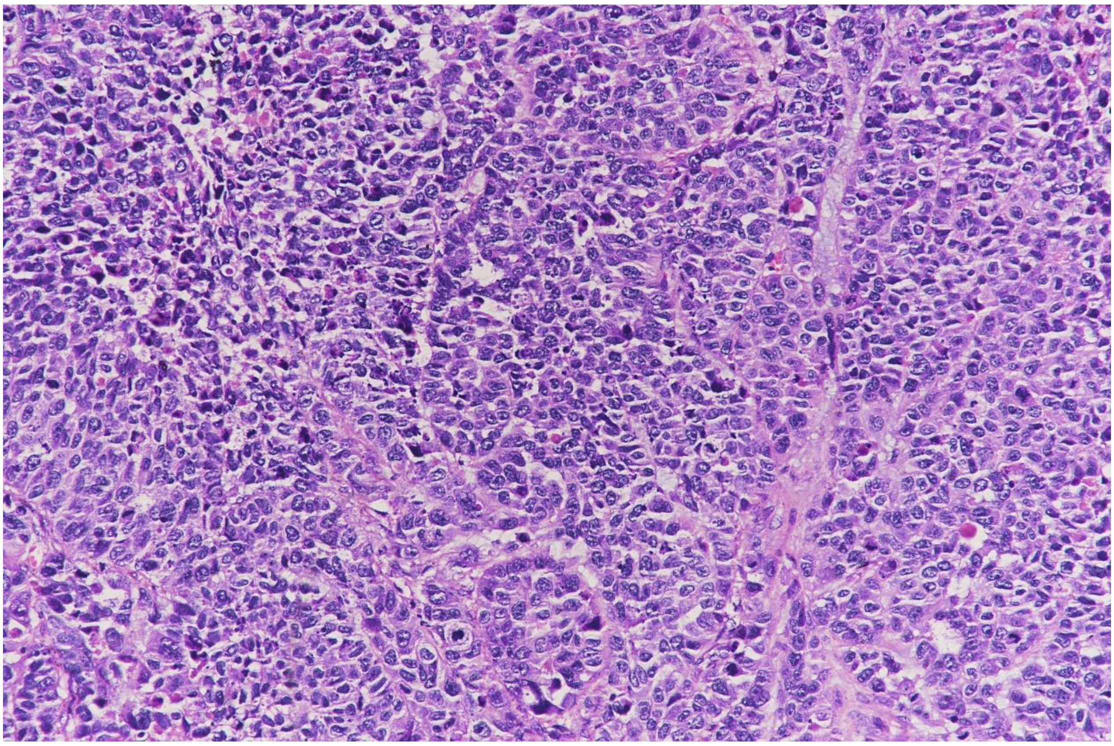

siCPNE3-1

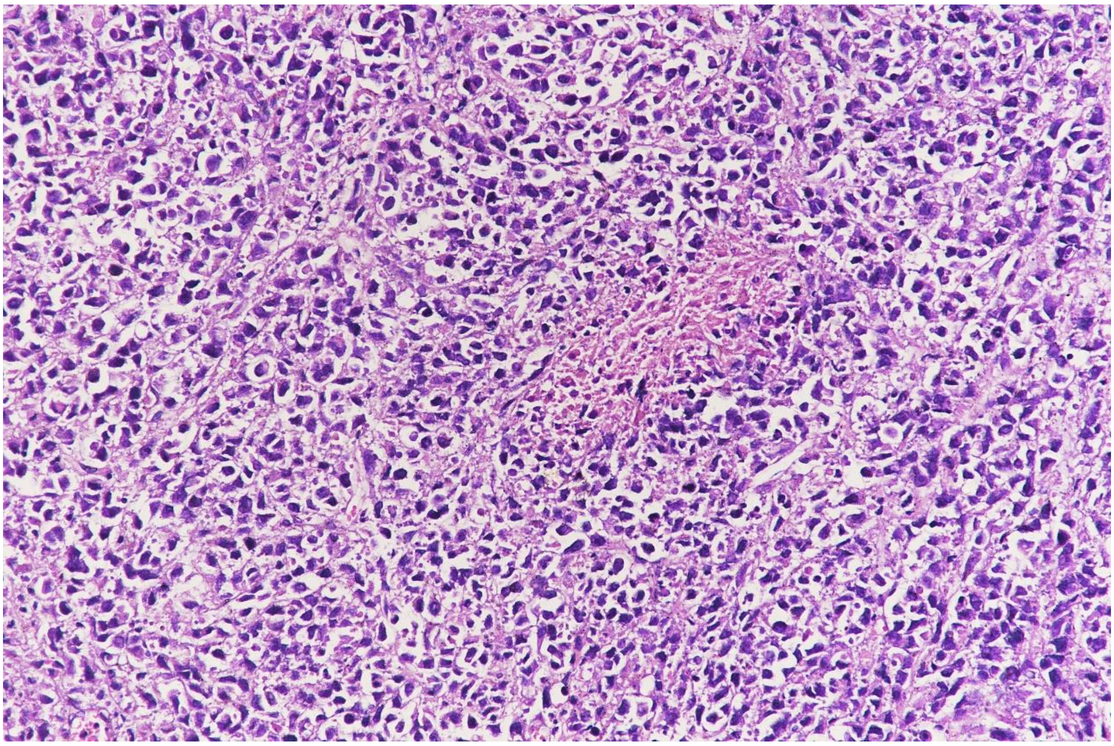

siCPNE3-2

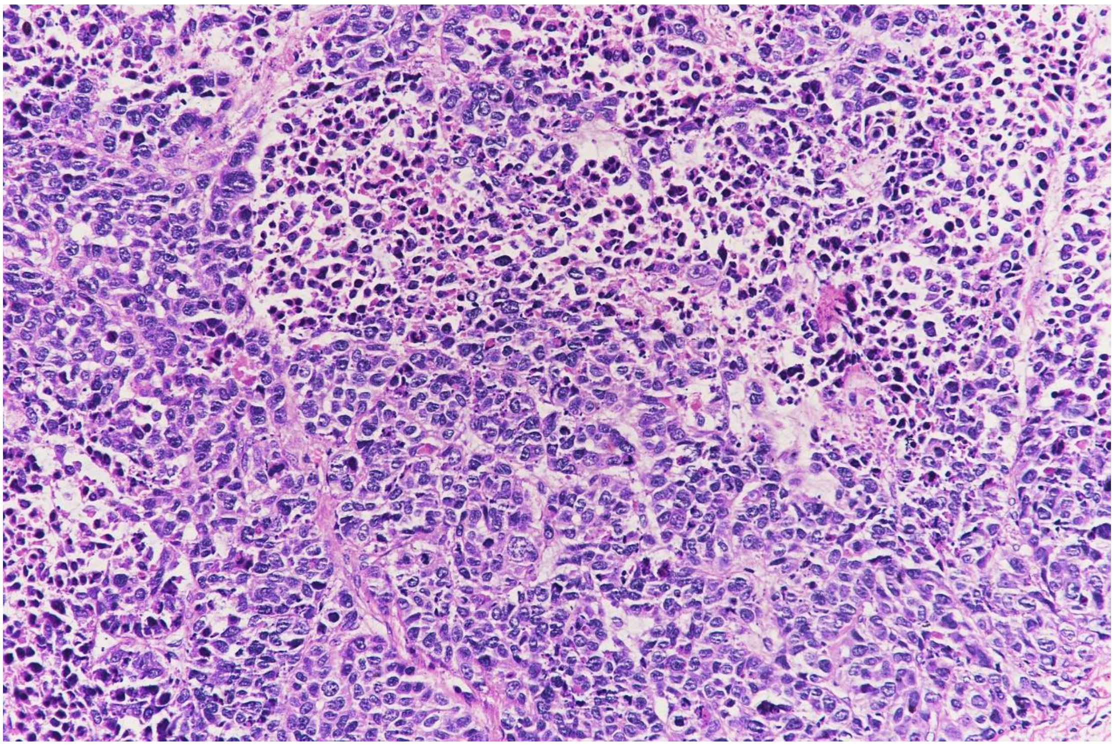

Supplement: Supplementary file 1 — Supplementary figures. [file jcav12p7277s1.pdf]
